# Genetic polymorphisms in *SLC5A2* are associated with clinical outcomes and dapagliflozin response in heart failure patients

**DOI:** 10.3389/fphar.2025.1539870

**Published:** 2025-04-28

**Authors:** Ahmed Essam Abou Warda, Rylie M. Flohr, Rania M. Sarhan, Mohamed Nabil Salem, Heba F. Salem, Ayman N. Moharram, Abdullah S. Alanazi, Christelle Lteif, Brian E. Gawronski, Leanne Dumeny, Tariq G. Alsahli, Khaled Elenizi, Bassem Zarif, Neven Sarhan, Julio D. Duarte

**Affiliations:** ^1^ Department of Clinical Pharmacy, Faculty of Pharmacy, October 6 University, Giza, Egypt; ^2^ Center for Pharmacogenomics and Precision Medicine and Department for Pharmacotherapy and Translational Research, University of Florida, Gainesville, FL, United States; ^3^ Department of Clinical Pharmacy, Faculty of Pharmacy, Beni-Suef University, Beni-Suef, Egypt; ^4^ Faculty of Pharmacy, Beni-Suef University, Beni-Suef, Egypt; ^5^ Department of Internal Medicine, Faculty of Medicine, Beni-Suef University, Beni-Suef, Egypt; ^6^ Department of Pharmaceutics and Industrial Pharmacy, Faculty of Pharmacy, Beni-Suef University, Beni-Suef, Egypt; ^7^ Program of Pharmaceutical Production, 6th October Technology University, Giza, Egypt; ^8^ Department of Critical Care Medicine, Faculty of Medicine, Cairo University, Giza, Egypt; ^9^ Department of Clinical Pharmacy, College of Pharmacy, Jouf University, Sakaka, Saudi Arabia; ^10^ Department of Pharmacology, College of Pharmacy, Jouf University, Sakaka, Saudi Arabia; ^11^ Department of Internal Medicine, College of Medicine, Prince Sattam bin Abdulaziz University, Alkharj, Saudi Arabia; ^12^ Department of Cardiology, National Heart Institute, Giza, Egypt; ^13^ Department of Clinical Pharmacy, Faculty of Pharmacy, Misr International University, Cairo, Egypt

**Keywords:** sodium-glucose transporter 2 inhibitors, heart failure, gene-drug interactions, dapagliflozin, single nuceotide polymorphism

## Abstract

**Background:**

Sodium-glucose cotransporter-2 inhibitors (SGLT2i) have emerged as promising therapeutics for heart failure (HF). Nevertheless, evidence supporting the mechanism of SGLT2i efficacy in HF patients is currently limited. Genetic variation in *SLC5A2* (encoding SGLT2) may influence HF progression and SGLT2i response, as well as inform potential SGLT2i mechanisms. Thus, this study investigated associations between *SLC5A2* variation and clinical outcomes in SGLT2i-naïve and dapagliflozin-treated HF cohorts.

**Methods:**

We analyzed two HF cohorts to identify variants associated with SGLT2i response pathways. Adjusted Cox proportional-hazard regression models were used to assess the effect of *SLC5A2* variation on a primary composite outcome of cardiovascular (CV) hospitalization or all-cause mortality in SGLT2i-naïve patients, and HF hospitalization or CV death in dapagliflozin-treated patients. The initial cohort comprised 327 American HF patients naïve to SGLT2i throughout the study. Subsequently, a prospective cohort study of 190 Egyptian SGLT2i-naïve HF patients treated with dapagliflozin was analyzed. In this cohort, SNPs in *UGT2B4* and *SLC2A1* were also investigated. Changes in NT-proBNP levels, KCCQ-12 scores, echocardiographic parameters, and eGFR throughout 6-month follow-up were tested with linear regression models as secondary outcomes.

**Results:**

In SGLT2i-naïve patients, rs3813008 (SLC5A2) was significantly associated with reduced risk of the composite outcome of all-cause death or hospitalization (HR = 0.65, 95% CI: 0.47–0.89, P = 0.008). In the dapagliflozin-treated cohort, rs3813008 was also associated with death or hospitalization, but with increased risk in treated patients (HR = 3.38, 95% CI: 1.35–8.42, P = 0.008).

**Conclusion:**

Our study suggests that *SLC5A2* variation is associated with clinical outcomes in SGLT2i-naïve and treated HF patients, warranting further investigation of *SLC5A2* and SGLT2i interactions.

## 1 Introduction

Heart failure (HF) is an expanding global health concern, affecting millions of individuals worldwide (Sapna et al., 2023). Despite recent advancements in pharmaceutical and device-based therapies, HF mortality and morbidity rates continue to rise (Fudim et al., 2021; Popa et al., 2022). The multifaceted pathophysiology of HF is likely influenced by genetics, environmental factors, and lifestyle choices, which may partially explain interindividual treatment response variability (Berthiaume et al., 2016; Ranek et al., 2022).

Sodium-glucose cotransporter two inhibitors (SGLT2i) have recently gained recognition as a promising HF treatment, reducing HF hospitalizations and cardiovascular (CV) deaths in clinical trials treating type 2 diabetes mellitus (T2DM) (Scholtes et al., 2019; Seferovic and Seferovic, 2020). Notably, dapagliflozin, an SGLT2i, has proven significant efficacy in lowering the risk of HF worsening in patients with reduced ejection fraction (HFrEF) and preserved ejection fraction (HFpEF), irrespective of T2DM status, as evidenced by the DAPA-HF and DELIVER trials, respectively (McMurrayet al., 2019; Solomon et al., 2022).

The precise mechanisms by which SGLT2i mitigate HF progression remain largely unknown. Proposed hypotheses suggest cardioprotective effects may stem from alleviating dysregulated processes such as inflammatory responses, cardiac remodeling, erythropoiesis, and sympathetic nervous system activity. SGLT2i may also improve cardiac energy production and regulate blood pressure (Lopaschuk and Verma, 2020; Dyck et al., 2022). Evidence further suggests direct effects on the heart through modulation of sodium-hydrogen exchanger 3 (NHE3) and glucose transporter 1 (GLUT1) function, thereby improving cellular communication and survival within cardiac tissue (Onishi et al., 2020; Packer, 2023). Dapagliflozin inhibits SGLT2, a transporter protein encoded by the *SLC5A2* gene. Mutations in *SLC5A2* can alter the expression, membrane localization, or function of SGLT2, potentially altering drug efficacy (Klen and Dolžan, 2021). During metabolism, dapagliflozin undergoes primarily glucuronidation mediated by the uridine 5′-diphosphate glucuronosyltransferase (UGT) isomer UGT1A9, with additional metabolism by UGT2B4 and cytochrome P450 3A4 (Marshall et al., 2021; Xu et al., 2023). Variations in the genes encoding these enzymes may alter dapagliflozin pharmacokinetics, leading to differences in plasma exposure and therapeutic response (Katzmann et al., 2021).

Presently, there is limited research regarding the genetic variations that may influence the safety and efficacy of SGLT2i in HF patients. This study aimed to identify associations between polymorphisms in putative SGLT2i response pathways and clinical outcomes in HF patients.

## 2 Materials and methods

### 2.1 Study design and cohorts

The SGLT2i-naïve cohort included patients with a diagnosis of HF (HFrEF or HFpEF) who were prospectively recruited from the University of Illinois at Chicago (UIC) cardiology clinics and followed from November 2001 to September 2015, with follow-up data recorded until November 2015. All patients were recruited and followed prior to SGLT2i use in HF and only patients not prescribed an SGLT2i were included in this study. Participants in the study provided written informed consent prior to their participation and all procedures were approved by the UIC Institutional Review Board (IRB# 2001-0345). Data was manually collected from the electronic health records and Social Security Death Index. Participants were censored if they were lost to follow-up or did not experience the event at the end of follow-up, and age was collected at the time of event or point of censorship. Medication use and blood samples for DNA isolation were collected as close to the study enrollment as possible. Throughout the follow-up period, treatment plans were monitored and adjusted at the discretion of the patient’s cardiologist. Additional details for this study population have been previously described (Lteif et al., 2020).

The dapagliflozin-treated cohort was an observational, prospective cohort study that was analyzed subsequently to the initial SGLT2i-naïve cohort and included 190 adult Egyptian patients (aged 18 years and older) with a confirmed diagnosis of HF. Patients were evaluated for HFrEF, left ventricular ejection fraction (LVEF) ≤45%, or HFpEF, LVEF >45%, originating from ischemic or non-ischemic etiology. The participants were classified within New York Heart Association (NYHA) functional classes II to IV and began dapagliflozin therapy at a dose of 10 mg once daily as part of their standard medical treatment. Enrollment took place between February 2023 and May 2024 at the HF Outpatient Clinics of the National Heart Institute (NHI) in Cairo, Egypt, ensuring a representative sample of the HF population typically treated at this facility. Exclusion criteria included a history of hypersensitivity to dapagliflozin, current or previous SGLT2i use, and patients in acute settings. Additionally, subjects with an estimated glomerular filtration rate (eGFR) less than 30 mL/min/1.73 m^2^, end-stage renal disease, or ALT and AST levels greater than three times the upper normal limit were excluded. The study received approval from the Research Ethics Committee of the Organization of Teaching Hospitals and Institutes (GOTHI), Cairo, Egypt (IRB# IHC00044), in accordance with the Helsinki Declaration’s rules and principles, and was registered at clinicaltrials.gov under the identifier NCT06201000. All participants provided written informed consent prior to data collection, blood sampling, and clinical evaluation.

### 2.2 Study protocol

Clinical data collected at baseline included assessments of age, sex, body mass index (BMI), medical/medication history, smoking status, and physical examination. The NYHA class and laboratory test results, including N-terminal pro b-type natriuretic peptide (NT-proBNP) and eGFR, were recorded. For dapagliflozin-treated patients, echocardiographic evaluations and quality-of-life assessments using the Kansas City Cardiomyopathy Questionnaire (KCCQ-12) were also performed. All treated patients were monitored monthly for HF hospitalization and CV death, with follow-up visits conducted 6 months after the initial visit in a dedicated outpatient clinic. At follow-up, echocardiographic evaluation, NT-proBNP, eGFR, and KCCQ-12 assessments were repeated.

The KCCQ-12 is a validated measure of health status for individuals with HF. It is composed of four domains (Physical Limitation, Symptom Frequency, Quality of Life, and Social Limitations) weighted on a scale from 0 (very poor health) to 100 (very good health). A summary score is calculated by averaging the four subdomain scores, with a five-point shift deemed clinically significant (Sauser et al., 2014; Spertus and Jones, 2015).

Cardiac structure and function were estimated using echocardiography. A thorough evaluation was performed on all dapagliflozin-treated patients utilizing an EPIQ echocardiography machine (Philips, Andover, MA, United states). LVEF was measured using the 2D transthoracic echocardiography in the biplane-modified Simpson’s method, and the LV wall thickness measurement was taken from the thickest segment of the LV. LVEF, fractional shortening (FS), left ventricular mass (LVM), and pulmonary artery systolic pressure (PASP) were measured according to the American Society of Echocardiography guidelines. The echocardiography was conducted by an independent cardiologist, not privy to the patient’s genetic information, and measurement discrepancies were resolved by consensus.

### 2.3 Genotyping

At enrollment, a peripheral venous blood sample was collected and stored at −80°C until DNA isolation in both cohorts. The SGLT2i-naïve cohort was genotyped using the Affymetrix Axiom PanAfrican Array (Thermo Fisher Scientific, Waltham, MA), as previously described (Guerra et al., 2022). Variants within 5 kilobases of the *SLC5A2* locus with global minor allele frequencies (MAF) greater than 0.05 were extracted with SNPnexus (https://www.snp-nexus.org/v4/) and pruned for linkage disequilibrium (*r* ^2^ > 0.6).

Dapagliflozin-treated patients were genotyped to explore genetic factors influencing drug efficacy, metabolism, and potential direct cardiac effects. First, four *SLC5A2* SNPs (rs3813008, rs4536493, rs9934336, rs9927250) significantly associated with clinical outcomes in the initial analysis of SGLT2i-naïve patients were selected. A literature search was conducted to identify additional variants. *SLC5A2* SNPs with previous associations reported with any SGLTi response were prioritized for inclusion. In addition, two SNPs rs72551330, in *UGT1A9,* and rs1080755, in *UGT2B4,* were selected given their predicted function in SGLT2i metabolism, which could putatively influence drug concentrations and toxicity (Xu et al., 2023). Additionally, previous research has suggested that SGLT2i might exert direct cardiac effects through glucose transporters, GLUT1 and GLUT4 (Packer, 2023). This warranted the inclusion of rs1385129, in *SLC2A1*, which encodes GLUT1 and may alter transporter function. Genotyping was performed using TaqMan^®^ SNP Genotyping Assays (Thermo Fisher Scientific) on an Applied Biosystems QuantStudio 12K Flex Real-Time PCR System. Predesigned TaqMan probes containing the necessary primers were used (Thermo Fisher Scientific, Inc.). Quality control was ensured by including negative controls and randomly selecting duplicate samples with a repeat rate of 20% to confirm concordance. To reduce the risk of bias, each blood sample was labeled with a unique identifier, preventing easy connection to clinical data during the genotyping process.

### 2.4 Clinical outcomes

Associations were first investigated between *SLC5A2* variants and the composite of CV hospitalization or all-cause mortality, as the primary outcome in an SGLT2i-naïve cohort. Cardiac-related hospitalization comprised of events such as stroke, myocardial infarction, unstable angina, and acute decompensated HF. All-cause mortality was further analyzed as a stand-alone secondary outcome.

In the dapagliflozin-treated patients, associations were assessed between *SLC5A2, UGT2B4* and *SLC2A1* variants and the risk of the composite of CV death or HF hospitalization during the first 6 months of dapagliflozin treatment. Changes in echocardiographic parameters, NT-proBNP, KCCQ-12, and eGFR before and after 6 months of follow-up following drug administration were secondary outcomes.

### 2.5 Statistical analysis

The Shapiro-Wilk test was used to test for normality of distribution, with the results shown as median and interquartile range (IQR). Categorical variables were displayed as percentages and frequencies. Cox proportional-hazard regression models were used to assess the hazard ratios (HR) and 95% confidence intervals (CIs) for the primary outcomes in both cohorts. The proportional hazard assumption was verified using scaled Schoenfeld residuals. Covariates for all models were selected *a priori* determined by clinical relevance, previously published literature, and data availability.

Associations between *SLC5A2* variants and the primary composite outcome (and secondarily on mortality alone) were evaluated in the SGLT2i-naïve cohort. The model was adjusted for age, sex, self-reported race, BMI, T2DM, NYHA class, smoking status, beta-blocker dosage level, ACE inhibitors (ACEi)/angiotensin receptor blocker (ARB) dose level, and aldosterone receptor antagonist (ARA) use. For variants with a MAF of 0.05 (the lower threshold for inclusion), our sample size would have 80% power to detect an effect size of 0.05 (small to moderate) with an α = 0.05.

A second model was used to assess the effect of SNPs in *SLC5A2, UGT2B4,* and *SLC2A1* on CV death or HF hospitalization in the dapagliflozin-treated cohort. This model included age, sex, NYHA class, smoking status, and the use of CV medications (ACEi/ARBs, angiotensin receptor-neprilysin inhibitors, beta-blockers, and mineralocorticoid receptor antagonists). Secondary clinical outcomes were evaluated with adjusted linear regressions with model covariates specific to the outcome. For variants with a MAF of 0.05, our sample size would have 80% power to detect an effect size of 0.08 (small to moderate) with an α = 0.05.

Initial analyses of all SNPs assumed an additive model (A/A vs A/a vs a/a). For SNPs that were rare (<5 homozygous variant patients), associations were re-analyzed assuming a dominant model (A/A vs A/a + a/a) to confirm prior associations were not driven by a very small number of homozygous patients. *P*-values ≤ 0.05 were considered statistically significant and all statistical analyses were performed using R (version 4.4.0) or PLINK (v1.9).

## 3 Results

### 3.1 Baseline characteristics of the study cohorts

The SGLT2i-naïve cohort included 327 HF patients with genotype data (Table 1). The cohort was racially diverse, consisting of 73.6% of African ancestry, 15.3% Latino, 10.1% Non-Latino European ancestry, and 0.9% Asian ancestry. The median age at event or censorship was 62.4 years. Most participants had HFrEF (89.3%) and were classified in NYHA functional classes II (30.1%) or III (42.0%). Throughout the observation period, 54.6% of patients experienced the primary composite outcome, with hospitalizations accounting for 84.3% of these events.

**TABLE 1:** Baseline characteristics of the SGLT2i-naïve and dapagliflozin-treated cohorts.

Variable	SGLT2i-naïve N = 327^a^	Dapagliflozin-treated N = 190^a^
Age at event/censor	62 (53, 71)	58 (50, 64)
Female	164 (50%)	64 (34%)
Self-reported race/ethnicity		
Asian	3 (0.9%)	0 (0%)
Black	240 (73%)	0 (0%)
Hispanic/Latino	51 (16%)	0 (0%)
Middle Eastern	0 (0%)	190 (100%)
Non-Latino White	33 (10%)	0 (0%)
BMI	33 (28, 39)	26 (24, 29)
NYHA Functional Class		
I	83 (26%)	0 (0%)
II	98 (30%)	100 (53%)
III	137 (42%)	78 (41%)
IV	5 (1.5%)	12 (6.3%)
HFpEF	35 (11%)	24 (13%)
Type 2 diabetes	164 (50%)	75 (39%)
Smoking Status		
Current smoker	138 (42%)	36 (19%)
Never smoker	132 (41%)	94 (49%)
Past smoker	55 (17%)	60 (32%)
Medications		
ACEI use	233 (72%)	86 (45%)
ARA use	63 (19%)	126 (66%)
ARB use	68 (21%)	17 (8.9%)
BB use	312 (96%)	152 (80%)

^a^ Median (IQR); n (%).

ACEI, angiotensin-converting enzyme inhibitor; ARA, aldosterone receptor antagonist; ARB, angiotensin receptor blocker; BB, beta-blockers; BMI, body mass index; HFpEF, heart failure with preserved ejection fraction; NYHA, new york heart association.

Of the 250 dapagliflozin-treated HF subjects enrolled in the study, 190 completed the 6-month follow-up and were included in the analysis. The remaining patients were lost to follow-up. The patients were of Middle Eastern descent, with a median age of 57.6 at event or censor (Table 1). At baseline, most patients had reduced LVEF (87.4%), with 47.9% of these cases attributed to ischemic etiology. Additionally, most were classified in NYHA class II (52.6%) or III (41.1%). Approximately 13.5% of the patients experienced the composite outcome during the study. Hospitalizations made up most of these events (88.0%).

### 3.2 Associations between genetic polymorphisms and outcomes in SGLT2i-naïve patients

**FIGURE 1:**
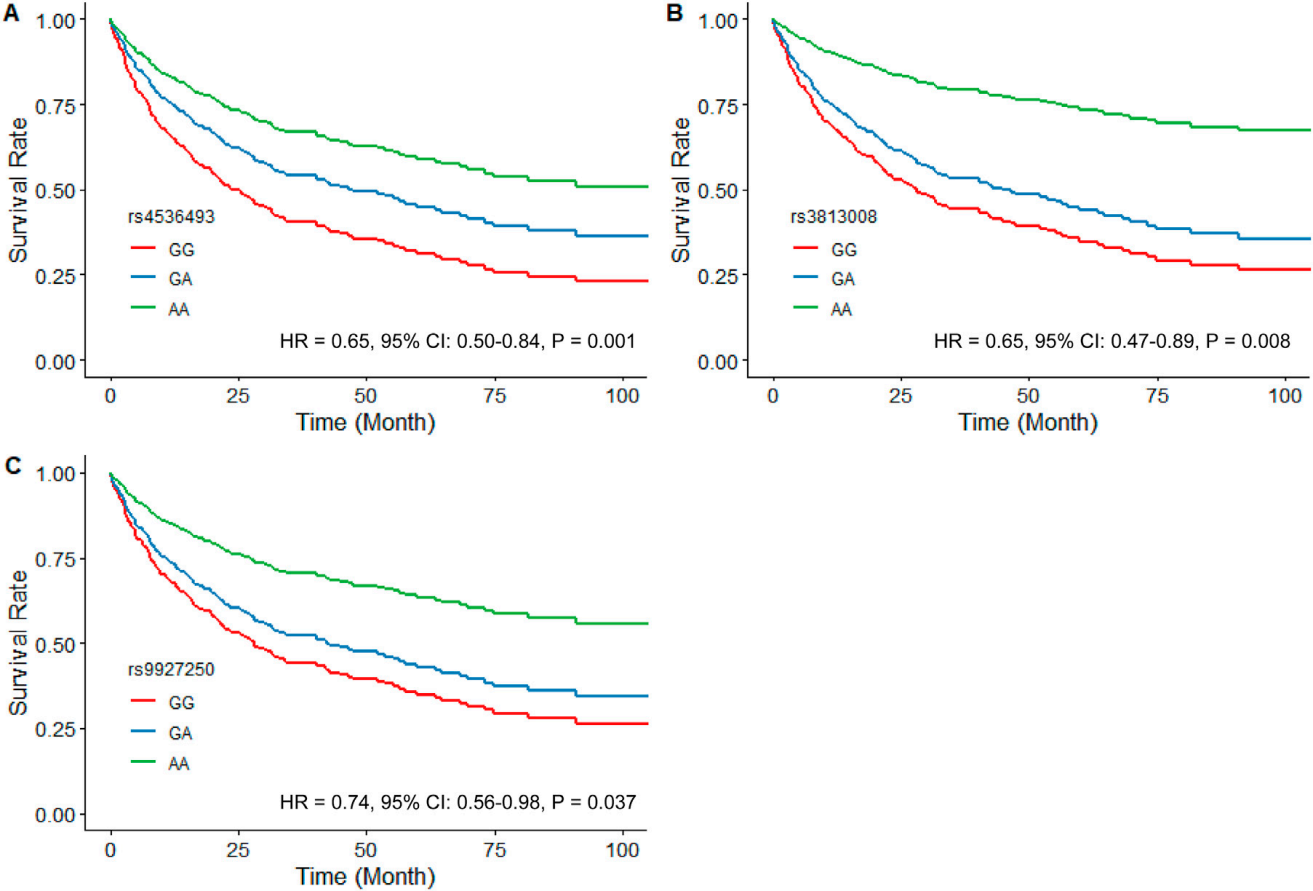
Estimated adjusted time-to-event survival curves of *SLC5A2* SNPs in SGLT2i-naïve patients. **(A)** rs4536493 (N = 179 for G/G, N = 116 for G/A, and N = 23 for A/A), **(B)** rs3813008 (N = 230 for G/G, N = 79 for G/A, and N = 9 for A/A), and **(C)** rs9927250 (N = 212 for G/G, N = 92 for G/A, and N = 14 for A/A). Lines denote the homozygous reference genotype (red), the heterozygous genotype (blue) and the homozygous variant genotype (green) respectively.

The analysis of *SLC5A2* SNPs in the SGLT2i-naïve HF cohort included 30 SNPs with similar MAFs to those previously reported in African populations. SNPs that violated Hardy-Weinberg equilibrium (rs11646054, rs138156533, and rs45437194) were excluded (Supplementary Table S1). Of the remaining 27 SNPs, rs4536493 (HR = 0.65, 95% CI: 0.50–0.84, *P* = 0.001), rs3813008 (HR = 0.65, 95% CI: 0.47–0.89, *P* = 0.008), and rs9927250 (HR = 0.74, 95% CI: 0.56–0.98, *P* = 0.037) were all associated with a reduced risk of the primary composite outcome of time to the first CV hospitalization or all-cause mortality (Figure 1).

Both rs4536493 (HR = 0.64, 95% CI: 0.43–0.94, *P* = 0.023) and rs3813008 (HR = 0.56, 95% CI: 0.33–0.94, P = 0.030) demonstrated a similar association with all-cause mortality. In contrast, rs34081766 (HR = 1.54, 95% CI: 1.08–2.19, *P* = 0.016) and rs9934336 (HR = 1.52, 95% CI: 1.07–2.16, *P* = 0.021) were significantly associated with an increased risk of mortality. Covariate-adjusted models met the proportional hazards assumption (global *P* > 0.30).

### 3.3 Associations between genetic polymorphisms and outcomes in dapagliflozin-treated patients

Variants in *SLC5A2, UGT2B4, SLC2A1,* and *UGT1A9* were in Hardy-Weinberg equilibrium, except rs72551330 in *UGT1A9* (*P* = 0.001) which was excluded from the final analysis (Supplementary Table S2). The repeat concordance rate was 100% for the genotype assays, except rs1385129 (97.9%) and rs9934336 (82.6%). The allele frequencies in the Egyptian cohort were close to those reported in the SGLT2i naïve cohort except for rs9934336.

**FIGURE 2:**
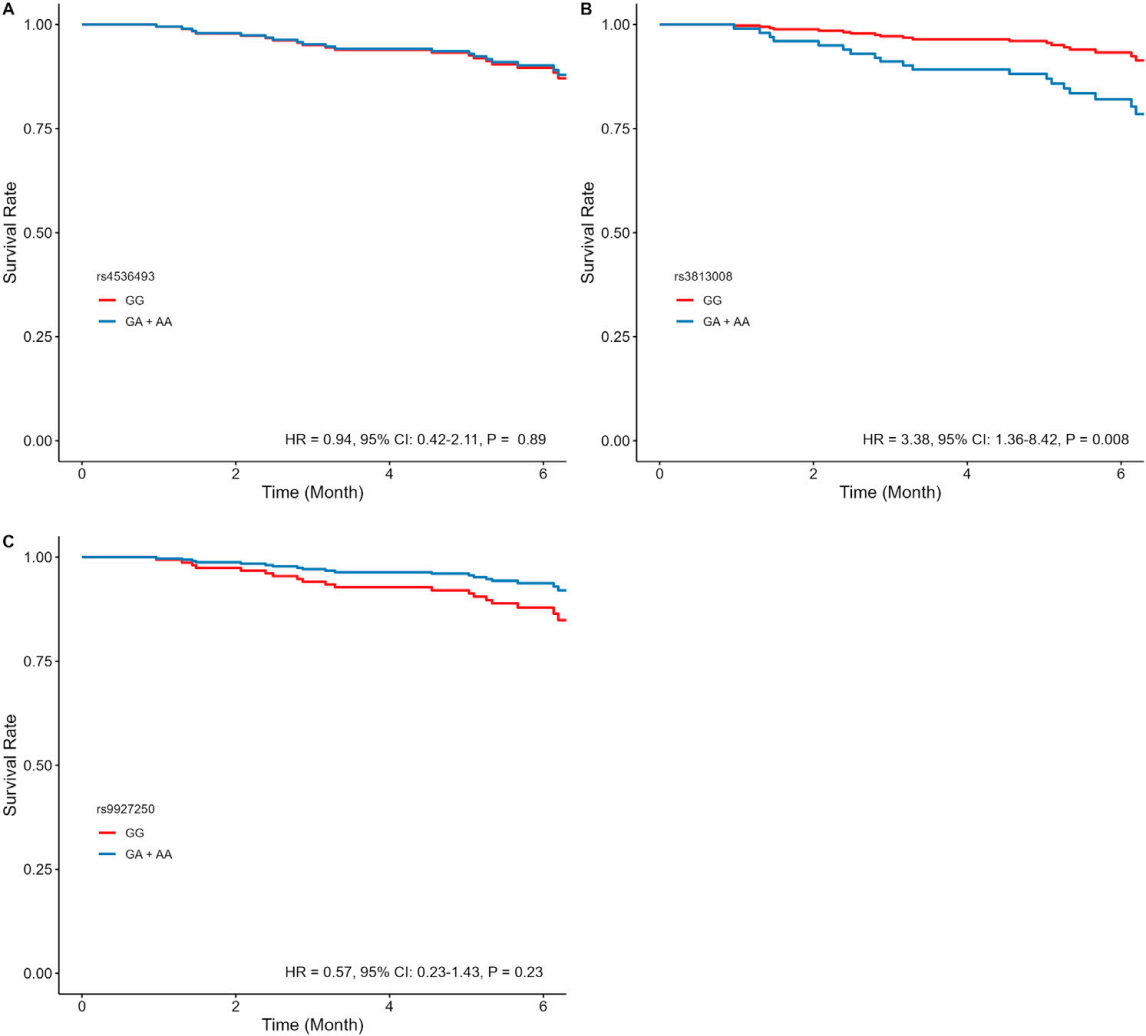
Estimated adjusted time-to-event survival curves of *SLC5A2* SNPs in dapagliflozin-treated patients. **(A)** rs4536493 (N = 86 for G/G, and N = 103 for G/A + A/A), **(B)** rs3813008 (N = 124 for G/G, and N = 66 for G/A + A/A), and **(C)** rs9927250 (N = 120 for G/G, and N = 68 for G/A+ A/A). Lines denote the homozygous reference genotype (red), and the heterozygous and homozygous variant genotypes (blue).

In dapagliflozin-treated patients, rs3813008 (in *SLC5A2*) was significantly associated with increased risk of CV mortality and HF hospitalizations (HR = 3.09, 95% CI: 1.30–7.39, *P* = 0.011). Adjusted time-to-event curves for the composite outcome were evaluated using the dominant model for rs4536493, rs3813008, and rs9927250, given the insufficient sample size of homozygous variant carriers. Rs3813008 remained significantly associated (HR = 3.38, 95% CI: 1.36–8.42, *P* = 0.008), while rs4536493 (HR = 0.94, 95% CI: 0.42–2.11, *P* = 0.89) and rs9927250 (HR = 0.57, 95% CI: 0.23–1.43, *P* = 0.23) were no longer significant (Figure 2). In a sensitivity analysis of patients with LVEF ≤45%, rs3813008 remained significantly associated with increased risk of the primary outcome (HR = 2.99, 95% CI: 1.16–7.71, *P* = 0.023; Supplementary Table S3). Due to the limited number of patients with EF >45% experiencing the outcome, a similar sensitivity analysis could not be performed.

As a secondary objective, we examined the association of dapagliflozin treatment with changes in echocardiographic parameters (LVEF, FS, LV Mass, PASP), NT-proBNP levels, and KCCQ-12 after 6 months of therapy. We also assessed potential associations with safety related to changes in renal function, estimated by eGFR, after 6 months of follow-up. No significant associations were observed for these variables in the overall cohort (Table 2). Sensitivity analyses for these outcomes were performed in patients with EF ≤ 45% and EF >45% (Supplementary Tables S4, S5). For patients with EF >45%, rs1080755(*UGT2B4*) and rs3813008 (*SLC5A2*) were significantly associated with improved KCCQ-12 scores (β = 12.06, *P* = 0.025, and β = 8.54, *P* = 0.042, respectively). Furthermore, rs9934336 (*SLC5A2*) was associated with elevated NT-proBNP levels for patients with EF >45% (β = 1,514.8, *P* = 0.045) (Supplementary Table S5). No serious adverse events related to SGLT2i treatment (such as diabetic ketoacidosis, Fournier’s gangrene, genital mycotic infections, or bone fractures) were reported for dapagliflozin-treated patients.

**TABLE 2:** Linear regression analysis for the associations between polymorphisms and secondary outcomes in the dapagliflozin-treated cohort.

SNP	Change in LVEF^a^ (%) ß (95%CI)	Change in FS^a^ (%) ß (95%CI)	Change in LVM^a^ (g) ß (95%CI)	Change in PASP^b^ (mmHg) ß (95%CI)	Change in NT-proBNP^a^ (pg/mL) ß (95%CI)	Change in KCCQ-12^c^ (Points) ß (95%CI)	Change in eGFR^d^ (ml/min/1.73m^2^) ß (95%CI)
***SLC5A2* rs3813008**	0.45 (−1.72–2.63)	−0.03 (−1.34–1.26)	21.83 (−10.06–53.73)	−0.55 (−4.36–3.26)	316.81 (−204.73–838.36)	3.11 (−2.18–8.41)	−5.14 (−10.55–0.27)
SE	1.11	0.66	16.27	1.94	266.1	2.70	2.76
P-value	0.682	0.955	0.182	0.777	0.237	0.250	0.064
***SLC5A2* rs4536493**	0.42 (−1.36–2.17)	0.12 (−0.95–1.15)	15.64 (−9.55–40.42)	−2.78 (−5.86–0.37)	196.21 (−208.98–601.40)	2.11 (−2.19–6.43)	−0.24 (−4.67–4.33)
SE	0.90	0.54	12.83	1.59	206.73	2.21	2.30
P-value	0.636	0.824	0.225	0.083	0.345	0.341	0.915
***SLC5A2* rs9934336**	−0.01 (−2.53–1.94)	0.01 (−1.46–1.21)	8.38 (−41.98–31.27)	0.17 (−3.35–4.38)	208.59 (−352.39–724.31)	1.37 (−3.81–7.26)	0.01 (−6.28–5.53)
SE	1.13	0.67	16.26	1.94	276.97	2.78	2.96
P-value	0.989	0.984	0.607	0.929	0.453	0.622	0.996
***SLC5A2* rs9927250**	1.73 (−28–3.66)	0.96 (−0.32–2.13)	20.92 (−9.40–45.80)	−2.64 (−6.02–0.94)	196.51 (−221.35–614.37)	0.63 (−4.31–5.48)	3.56 (−1.32–8.29)
SE	1.01	0.6	14.11	1.78	213.20	2.51	2.46
P-value	0.090	0.114	0.141	0.139	0.359	0.800	0.149
***UGT2B4* rs1080755**	−1.08 (−3.11–1.40)	−0.60 (−1.86–0.83)	−21.19 (−54.04–10.93)	−2.99 (−6.56–1.37)	−105.40 (−673.01–462.21)	0.31 (−5.80–5.49)	0.64 (−6.38–5.74)
SE	1.19	0.71	16.65	2.12	289.60	3.00	3.19
P-value	0.369	0.400	0.205	0.160	0.716	0.916	0.840
***SLC2A1* rs1385129**	0.10 (−1.74–2.00)	−0.004 (−1.11–1.13)	3.50 (−23.89–31.13)	2.07 (−1.38–5.42)	37.50 (−447.10–522.11)	−1.97 (−6.54–2.56)	−0.59 (−5.57–4.18)
SE	0.96	0.57	14.12	1.74	247.25	2.33	2.49
P-value	0.910	0.993	0.804	0.238	0.879	0.399	0.810
N	172	172	142	158	90	180	164

eGFR, estimated Glomerular Filtration Rate; FS, fractional shortening; KCCQ, kansas city cardiomyopathy questionnaire; LVEF, left ventricular ejection fraction; LVM, left ventricular mass; NT-proBNP, N-terminal pro–B-type natriuretic peptide; N, number of patients involved in each analysis; PASP, pulmonary artery systolic pressure; SE, standard error.

^a^ Model adjusted for Age, Sex, LVEF, range, ACE/ARB/ARNI, BB, MRA.

^b^ Model adjusted for Age, Sex, Atrial fibrillation, LVEF, range.

^c^ Model adjusted for Age, Sex, NYHA, smoking status.

^d^ Model adjusted for Age, Sex, LVEF, range, Furosemide, Torsemide, MRA, ACE/ARB/ARNI.

## 4 Discussion

Our study investigated the impact of genetic variations in *SLC5A2* (encoding SGLT2) on clinical outcomes in SGLT2i-treated and untreated HF patients. We also explored variants putatively involved in SGLT2i metabolism (*UGT2B4*) and glucose transport (*SLC2A1*) in dapagliflozin-treated HF patients. To our knowledge, this was the first study to investigate the interindividual genetic effect on the safety and efficacy of SGLT2i in HF patients. Our study demonstrated that the rs3813008 variant in *SLC5A2* was associated with cardiac-related hospitalization or mortality, with effects seeming to differ by treatment status. In SGLT2i-naïve patients, rs3813008 appeared protective, while dapagliflozin-treated carriers exhibited increased CV risk. These findings suggest that rs3813008 may confer a greater survival benefit in SGLT2i-naïve HF patients but could impact dapagliflozin efficacy. Genetic variation has previously been reported to modify protein and transporter function in dapagliflozin’s mechanism of action (Wang et al., 2024). However, rs3813008 is an intronic SNP, so the mechanism by which it may affect SGLT2 function is unclear. One potential explanation is that the SNP diminishes SGLT2 function, acting almost as a natural SGLT2i. Thus, when these patients take a medication to block the transporter, they derive little (or no) additional benefit compared to other patients. Additional functional studies of this variant should elucidate the possible mechanism by which this SNP could affect outcomes in HF.

While this is the first report of rs3813008 associations in the context of SGLT2i-treated HF patients, the SNP has been studied in other patient populations. A prospective study in patients with high CV risk indicated that rs3813008 was not associated with incidence of CV events, such as atherosclerosis and coronary artery disease, but did not include HF (Drexel et al., 2019). A second study conducted in United Kingdom Biobank and LUdwigshafen RIsk and Cardiovascular Health (LURIC) patients investigated *SLC5A2* variants expressed in any tissue (*P* < 0.05) and their association with HF incidence (both HFrEF and HFpEF) (Drexel et al., 2019; Katzmann et al., 2021). *SLC5A2* variants, including rs3813008 and rs9934336, were associated with a trend toward reduced HF occurrence. Furthermore, their genetic risk analysis for *SLC5A2* variants showed decreased HF risk for each additional allele inherited, with a more pronounced effect in patients without T2DM or coronary artery disease.

In addition to reduced risk of HF incidence, findings from the LURIC study indicated that the rs9934336 variant was associated with slightly lower NT-proBNP levels (Katzmann et al., 2021). In comparison, our subgroup analyses in patients with EF >45% treated with dapagliflozin found that rs9934336 was nominally associated with increased NT-proBNP levels. However, the LURIC study did not conduct a subgroup analysis evaluating SNP associations with NT-proBNP responses. These findings suggest that the pharmacogenetic effects of *SLC5A2* variants may be context-dependent, potentially influenced by EF class or treatment status. This emphasizes the need for further research given the currently limited evidence available.

Other *SLC5A2* variants studied in our cohorts, rs4536493 and rs9927250, exhibited lower risks of the composite outcome in SGLT2i-naïve patients, while rs9934336 and rs34081766 were associated with higher risks. However, similar associations were not observed in dapagliflozin-treated patients. This could be because the dapagliflozin cohort was smaller or because these variants may have a weaker influence once patients are treated with an SGLT2i or because of differences in minor allele frequencies of some SNPs between the two cohorts. Additional validation of these associations is needed.

Our sensitivity analyses between HF subgroups in dapagliflozin-treated patients revealed that rs3813008 (*SLC5A2*) and rs1080755 (*UGT2B4*) were associated with improved KCCQ-12 scores in patients with EF >45%. The UGT enzyme is a major component in dapagliflozin metabolism, contributing to individual variability in pharmacokinetics, pharmacodynamics, and treatment response of SGLT2i (Kaur et al., 2021; Klen and Dolžan, 2021). A study conducted by Francke et al. suggested that carrying the mutant allele rs1080755 in *UGT2B4* was associated with reduced glucuronidation rates, thereby increasing SGLT2i plasma concentrations compared to the wild-type allele (Francke et al., 2015). However, Kadric et al. reported no difference in SGLT2i plasma concentrations between wild-type and minor *UGT2B4* allele carriers, despite reduced O-glucuronide metabolite levels in mutant allele carriers (Kadrić et al., 2021). Our results also support a genome-wide study in the Kazakh population, proposing this SNP as a potential pharmacogenetic marker for dapagliflozin dosing (Svyatova et al., 2023). Further research of rs1080755 should be performed to identify whether clinical implications exist in dapagliflozin-treated HF patients.

GLUT1 (*SLC2A1*) and GLUT4 (*SLC2A4*) are glucose transporters expressed in cardiac tissue (Bertrand et al., 2020). While GLUT4 is insulin-regulated and predominantly expressed in healthy cardiac tissue, GLUT1-mediated glucose transport favors glycolysis and facilitates the influx of excess unregulated glucose. The bias becomes particularly pronounced when mitochondrial function is compromised, which frequently occurs in HF patients (Fillmore et al., 2018; Lopaschuk et al., 2021). A recent review suggests that SGLT2i exerts direct effects on cardiac muscle by inhibiting GLUT1 and reducing anaerobic glycolysis in cardiomyocytes, enhancing glucose oxidation (Packer, 2023). Literature has also suggested that rs1385129 (in *SLC2A1*) might increase susceptibility to nephropathy (Cui et al., 2012; Stefanidis et al., 2018). We found no association with rs1385129 in dapagliflozin-treated patients.

A major strength of our study is that we used two independent cohorts, including a diverse mix of HF patients, and that we tested associations both with and without SGLT2i treatment. Furthermore, this study paves the way for future research on gene-drug interactions in SGLT2i, offering new insights into personalized treatment approaches. Additionally, the genetic similarity between the predominantly African and Latino SGLT2i-naïve cohort and the Egyptian dapagliflozin-treated cohort enhances the study’s generalizability of potential pharmacogenetic associations. However, there are also several limitations to our study. First, the relatively small sample sizes of our cohorts likely affected the statistical power of our study, reducing our ability to detect smaller effect sizes. The sample size of the dapagliflozin-treated cohort was restricted in part due to the failure of some patients to follow up within the study window, introducing a potential bias of excluding patients who were unable to maintain their follow-up visits. While acknowledging this limitation, the meticulous collection of our clinical data likely enhanced the reliability of our findings. Second, population differences between two cohorts, including certain allele frequencies may act as confounders and affect the results. Finally, follow-up duration varied significantly between the two cohorts, with the untreated cohort having a longer follow-up, providing more stable hazard ratio estimates. In contrast, the shorter follow-up in the treated cohort may have amplified acute effects. In addition, our genotyping concordance rate for rs9934336 was lower than expected, which may have limited our ability to detect weaker associations related to that SNP. Despite these limitations, our study provides novel insights into potential SNPs that may contribute to differential responses to SGLT2i treatment, underscoring the need for larger, long-term studies to validate these associations and further investigate their role in personalized treatment strategies.

## 5 Conclusion

Genetic variation within the SGLT2i metabolic pathway may be clinically significant for HF patients. Our findings for rs3813008 in *SLC5A2* indicated differing results depending on SGLT2i treatment status, suggesting a possible interaction with dapagliflozin drug response. Other variants in *SLC5A2* and *UGT2B4* may also participate in SGLT2i metabolism and influence clinical outcomes. Comprehensive research into gene-drug effects in SGLT2i-treated HF patients could support personalized therapy. However, additional studies in larger studies are warranted to further validate these findings.

## Data Availability

The original contributions presented in the study are included in the article and Supplemental Material, further inquiries can be directed to the corresponding author.
