# Supplementary material for: Genetic polymorphisms in SLC5A2 are associated with clinical outcomes and dapagliflozin response in heart failure patients
Source: Front Pharmacol. 2025 Apr 28;16:1539870. doi: 10.3389/fphar.2025.1539870 (PMC12066643; doi:10.3389/fphar.2025.1539870)
Supplement: Supplementary file 1 [file Table1.docx]

**Genetic Polymorphisms in *SLC5A2* are Associated with Clinical Outcomes and Dapagliflozin** **Response in Heart Failure Patients**

Ahmed Essam Abou Warda ^1^, Rylie M. Flohr ^2^, Rania M. Sarhan ^3^, Mohamed Nabil Salem ^4^, Heba F. Salem ^5,6^, Ayman N. Moharram ^7^, Abdullah S Alanazi ^8^, Christelle Lteif ^2^, Brian E. Gawronski ^2^, Leanne Dumeny ^2^, Tariq G. Alsahli ^9^, Khaled Elenizi ^10^, Bassem Zarif ^11^, Neven Sarhan ^12^, Julio D. Duarte ^2, *^

1. Department of Clinical Pharmacy, Faculty of Pharmacy, October 6 University, Giza, Egypt

2. Center for Pharmacogenomics and Precision Medicine and Department for Pharmacotherapy and Translational Research, University of Florida, Gainesville, FL, USA

3. Department of Clinical Pharmacy, Faculty of Pharmacy, Beni-Suef University, Beni-Suef, Egypt

4. Department of Internal Medicine, Faculty of Medicine, Beni-Suef University, Beni-Suef, Egypt

5. Department of Pharmaceutics & Industrial Pharmacy, Faculty of Pharmacy, Beni-Suef University, Beni-Suef, Egypt

6. Program of Pharmaceutical Production, 6^th^ October Technology University, Giza, Egypt

7. Department of Critical Care Medicine, Faculty of Medicine, Cairo University, Giza, Egypt

8. Department of Clinical Pharmacy, College of Pharmacy, Jouf University, Sakaka, Saudi Arabia

9. Department of Pharmacology, College of Pharmacy, Jouf University, Sakaka, Saudi Arabia

10. Department of Internal Medicine, College of Medicine, Prince Sattam bin Abdulaziz University, Alkharj, Saudi Arabia

11. Department of Cardiology, National Heart Institute, Giza, Egypt

12. Department of Clinical Pharmacy, Faculty of Pharmacy, Misr International University, Cairo, Egypt

* Corresponding author: Julio D Duarte, Address: HSC PO Box 100486, Gainesville, FL 32610-0486, USA, Email: [juliod@cop.ufl.edu](mailto:juliod@cop.ufl.edu)

**Funding:** The research was funded by the Deputyship for Research & Innovation, Ministry of Education, Riyadh, Saudi Arabia through grant number 223202.

**Conflict of interest:** The authors declared no competing interests.

**Keywords**: Sodium-Glucose Transporter 2 Inhibitors, Heart Failure, Gene-Drug Interactions, dapagliflozin

**Supplemental information**

**Table S1 Distribution of the *SLC5A2* polymorphisms among the SGLT2i-naïve cohort**

| SNP | Reference (A1) | Alternate (AX) | A1/A1 Count | A1/AX Count | AX/AX Count | MAF | HWE P-value |
| --- | --- | --- | --- | --- | --- | --- | --- |
| rs4536493 | G | A | 183 | 121 | 23 | 0.255 | 0.662 |
| rs11150626 | T | C | 185 | 122 | 20 | 0.248 | 1.000 |
| rs9934336 | G | A | 187 | 121 | 19 | 0.243 | 1.000 |
| rs34081766 | C | A | 189 | 119 | 19 | 0.240 | 1.000 |
| rs11646054 | G | C | 224 | 80 | 23 | 0.193 | 0.000 |
| rs9927250 | G | A | 216 | 97 | 14 | 0.191 | 0.474 |
| rs113024267 | T | C | 228 | 84 | 15 | 0.174 | 0.055 |
| rs3813008 | G | A | 235 | 83 | 9 | 0.154 | 0.670 |
| rs73538351 | G | A | 251 | 72 | 4 | 0.122 | 0.800 |
| rs28687124 | A | G | 265 | 58 | 4 | 0.101 | 0.554 |
| rs9927039 | T | C | 265 | 58 | 4 | 0.101 | 0.554 |
| rs3116150 | G | A | 273 | 51 | 3 | 0.087 | 0.723 |
| rs138156533 | C | G | 280 | 41 | 6 | 0.081 | 0.012 |
| rs45437194 | C | G | 282 | 39 | 6 | 0.078 | 0.008 |
| rs45601239 | A | G | 281 | 42 | 4 | 0.076 | 0.106 |
| rs34303653 | T | C | 279 | 46 | 2 | 0.076 | 1.000 |
| rs115146436 | A | C | 289 | 37 | 1 | 0.060 | 1.000 |
| rs138578171 | A | G | 290 | 36 | 1 | 0.058 | 1.000 |
| rs74015544 | C | T | 295 | 31 | 1 | 0.050 | 0.573 |
| rs115034334 | C | T | 297 | 29 | 1 | 0.047 | 0.526 |
| rs116220123 | C | T | 297 | 29 | 1 | 0.047 | 0.526 |
| rs45500597 | C | T | 301 | 25 | 1 | 0.041 | 0.429 |
| rs74015546 | T | C | 303 | 24 | 0 | 0.037 | 1.000 |
| rs149498984 | T | C | 306 | 19 | 2 | 0.035 | 0.052 |
| rs77850731 | C | T | 306 | 20 | 1 | 0.034 | 0.306 |
| rs116337709 | T | C | 308 | 18 | 1 | 0.031 | 0.259 |
| rs3116149 | G | A | 307 | 20 | 0 | 0.031 | 1.000 |
| rs111332953 | C | T | 313 | 14 | 0 | 0.021 | 1.000 |
| rs3813007 | A | T | 315 | 12 | 0 | 0.018 | 1.000 |
| rs78899202 | C | A | 320 | 7 | 0 | 0.011 | 1.000 |

HWE, Hardy Weinberg equilibrium; MAF, Minor allele frequency; SNP, Single nucleotide polymorphism

**Table S2 Distribution of the gene polymorphisms among the dapagliflozin-treated cohort**

| **Gene** | **SNP** | **n** | **Genotype** | **n per genotype (%)** | **Genotype Frequency** | **MAF** | **HWE**  **P- Value** |
| --- | --- | --- | --- | --- | --- | --- | --- |
| ***SLC5A2*** | **rs3813008** | 190 | GG | 124 | 65.2% | 0.184 | 0.085 |
|  |  |  | GA | 62 | 32.6% |  |  |
|  |  |  | AA | 4 | 2.1% |  |  |
| **SLC5A2** | **rs4536493** | 189 | GG | 86 | 45.5% | 0.272 | 0.186 |
|  |  |  | GA | 86 | 45.5% |  |  |
|  |  |  | AA | 17 | 8.9% |  |  |
| **SLC5A2** | **rs9934336** | 189 | GG | 134 | 70.8% | 0.145 | 0.072 |
|  |  |  | GA | 49 | 25.9% |  |  |
|  |  |  | AA | 6 | 3.1% |  |  |
| **SLC5A2** | **rs9927250** | 188 | GG | 120 | 63.8% | 0.180 | 0.103 |
|  |  |  | GA | 60 | 31.9% |  |  |
|  |  |  | AA | 8 | 9.09% |  |  |
| **UGT1A9** | **rs72551330** | 190 | TT | 189 | 99.4% | 0.002 | 0.001 |
|  |  |  | CT | 1 | 0.52% |  |  |
|  |  |  | CC | 0 | 0% |  |  |
| **UGT2B4** | **rs1080755** | 188 | GG | 141 | 75% | 0.125 | 0.052 |
|  |  |  | GA | 44 | 23.4% |  |  |
|  |  |  | AA | 3 | 1.5% |  |  |
| **SLC2A1** | **rs1385129** | 189 | GG | 105 | 55.5% | 0.222 | 0.140 |
|  |  |  | GA | 73 | 38.6% |  |  |
|  |  |  | AA | 11 | 5.8% |  |  |

HWE, Hardy Weinberg equilibrium; MAF, Minor allele frequency; SNP, Single nucleotide polymorphism

**Table S3 Cox proportional hazards regression analysis for associations between polymorphisms with composite outcomes among patients with EF ≤ 45% in the dapagliflozin-treated cohort**

| **SNP** | **Adjusted Hazard ratio** | **95% Confidence Interval** | **P-value** ^a^ |
| --- | --- | --- | --- |
| **SLC5A2**  **rs3813008** | 2.99 | 1.16 - 7.71 | 0.023 * |
| **SLC5A2**  **rs4536493** | 1.05 | 0.53 - 2.06 | 0.876 |
| **SLC5A2**  **rs9934336** | 1.07 | 0.43 - 2.64 | 0.872 |
| **SLC5A2**  **rs9927250** | 0.79 | 0.35 - 1.78 | 0.580 |
| **UGT2B4**  **rs1080755** | 1.23 | 0.47 - 3.21 | 0.670 |
| **SLC2A1**  **rs1385129** | 0.62 | 0.25 - 1.53 | 0.306 |

a, Model adjusted for age, sex, NYHA class, smoking status, and CV medications (ACE inhibitors/ARBs, angiotensin receptor-neprilysin inhibitors, beta-blockers, and mineralocorticoid receptor antagonists); *, Significant p-value less than 0.05

**Table S4 Linear regression analysis for the associations between polymorphisms and secondary outcomes among patients with EF ≤ 45% in the dapagliflozin-treated cohort**

| **SNP** | **Change in LVEF** ^a^  **(%)**  **ß (95%CI)** | **Change in FS** ^a^  **(%)**  **ß (95%CI)** | **Change in LVM** ^a^  **(g)**  **ß (95%CI)** | **Change in PASP** ^b^  **(mmHg)**  **ß (95%CI)** | **Change in**  **NT-proBNP** ^a^  **(pg/mL)**  **ß (95%CI)** | **Change in KCCQ-12** ^c^  **(Points)**  **ß (95%CI)** | **Change in eGFR** ^d^  **(ml/min/**  **1.73m2)**  **ß (95%CI)** |
| --- | --- | --- | --- | --- | --- | --- | --- |
| ***SLC5A2***  **rs3813008** | 0.37 (-2.13 - 2.88) | -0.26 (-1.71 – 1.18) | 22.69 (-14.21- 59.60) | -0.04 (-4.66- 4.28) | 353.66 (-303.20 – 1010.53) | 2.08 (-4.25 - 8.43) | -4.00 (-10.20 -2.18) |
| **SE** | 1.27 | 0.73 | 18.83 | 2.22 | 335.14 | 3.23 | 3.15 |
| **P-value** | 0.770 | 0.719 | 0.231 | 0.982 | 0.295 | 0.519 | 0.206 |
| ***SLC5A2***  **rs4536493** | 0.43 (-1.54 - 2.37) | 0.17 (-0.97 – 1.28) | 19.32 (-9.04- 47.01) | -2.60 (-6.10 - 0.94) | 219.90 (-265.11 – 704.92) | 1.79 (-3.12 - 6.75) | 0.82 (-4.09- 5.87) |
| **SE** | 1.00 | 0.58 | 14.39 | 1.81 | 247.46 | 2.53 | 2.55 |
| **P-value** | 0.663 | 0.768 | 0.182 | 0.153 | 0.377 | 0.478 | 0.747 |
| ***SLC5A2***  **rs9934336** | 0.92 (-1.79 – 3.05) | 0.56 (-0.98 – 1.82) | 12.67 (-21.29- 50.61) | 1.34 (-2.39 – 6.18) | 130.13 (-491.41-707.04) | 2.11 (-3.52 – 8.59) | -0.09 (-6.66 - 5.65) |
| **SE** | 1.21 | 0.70 | 17.91 | 2.15 | 308.13 | 3.04 | 3.08 |
| **P-value** | 0.451 | 0.427 | 0.481 | 0.532 | 0.674 | 0.487 | 0.974 |
| ***SLC5A2***  **rs9927250** | 1.72 (-0.40 - 3.79) | 1.06 (-0.16 – 2.25) | 23.75 (-9.09- 50.17) | -2.37 (-6.05 – 1.60) | 187.55 (-275.68 – 650.78) | 1.97 (-3.49 – 7.30) | 3.44 (-1.77 - 8.45) |
| **SE** | 1.08 | 0.62 | 15.19 | 1.96 | 236.34 | 2.78 | 2.62 |
| **P-value** | 0.113 | 0.093 | 0.121 | 0.228 | 0.430 | 0.479 | 0.191 |
| ***UGT2B4***  **rs1080755** | -0.16 (-2.44- 2.49) | -0.20 (-1.56 -1.29) | -19.07 (-55.69 -16.87) | -2.98 (-7.05 -1.81) | -85.46 (-762.36 – 591.43) | -1.81 (-8.55 – 4.10) | 1.53 (-5.97 -6.77) |
| **SE** | 1.32 | 0.76 | 18.30 | 2.38 | 345.36 | 3.39 | 3.37 |
| **P-value** | 0.901 | 0.793 | 0.307 | 0.213 | 0.805 | 0.593 | 0.650 |
| ***SLC2A1***  **rs1385129** | -0.14 (-2.13- 1.88) | -0.23 (-1.37-0.94) | -0.95 (-30.85 -29.53) | 2.59 (-1.11 -6.24) | -17.1 (-576.74 – 542.53) | -2.27 (-7.38 - 2.81) | 0.68 (-4.71 -5.84) |
| **SE** | 1.03 | 0.59 | 15.51 | 1.89 | 285.53 | 2.61 | 2.70 |
| **P-value** | 0.887 | 0.698 | 0.951 | 0.172 | 0.952 | 0.386 | 0.799 |
| **N** | 149 | 149 | 123 | 139 | 76 | 158 | 151 |

eGFR, estimated Glomerular Filtration Rate; FS, Fractional shortening; KCCQ, Kansas City Cardiomyopathy Questionnaire; LVEF, left ventricular ejection fraction; LVM, left ventricular mass; NT-proBNP, N-terminal pro–B-type natriuretic peptide; N, Number of patients involved in each analysis; PASP, Pulmonary artery systolic pressure; SE, Standard error

a, Model adjusted for Age, Sex, LVEF range, ACE/ARB/ARNI, BB, MRA; b, Model adjusted for Age, Sex, Atrial fibrillation, LVEF range; c, Model adjusted for Age, Sex, NYHA, Smoking status; d, Model adjusted for Age, Sex, LVEF range, Furosemide, Torsemide, MRA, ACE/ARB/ARNI

**Table S5 Linear regression analysis for the associations between polymorphisms and secondary outcomes among patients with EF > 45% in the dapagliflozin-treated cohort**

| **SNP** | **Change in LVEF** ^a^  **(%)**  **ß (95%CI)** | **Change in FS** ^a^  **(%)**  **ß (95%CI)** | **Change in LVM** ^a^  **(g)**  **ß (95%CI)** | **Change in PASP** ^b^  **(mmHg)**  **ß (95%CI)** | **Change in**  **NT-proBNP** ^a^  **(pg/mL)**  **ß (95%CI)** | **Change in KCCQ-12** ^c^  **(Points)**  **ß (95%CI)** | **Change in eGFR** ^d^  **(ml/min/**  **1.73m2)**  **ß (95%CI)** |
| --- | --- | --- | --- | --- | --- | --- | --- |
| ***SLC5A2***  **rs3813008** | 1.02 (-5.46 – 7.51) | 0.77 (-3.77 – 5.33) | -15.34 (-91.03- 60.33) | -2.31 (-9.22 – 5.13) | -447.17 (-1328.34 – 433.99) | 8.54 (0.89 – 16.19) | -7.46 (-20.96 -7.01) |
| **SE** | 3.31 | 2.32 | 38.61 | 3.47 | 449.59 | 3.90 | 8.32 |
| **P-value** | 0.761 | 0.743 | 0.698 | 0.513 | 0.353 | 0.042* | 0.391 |
| ***SLC5A2***  **rs4536493** | 0.76 (-4.64 – 6.16) | -0.01 (-3.81 – 3.79) | -41.33 (-96.48- 13.81) | -2.18 (-10.01 – 4.01) | -370.62 (-1066.18 – 324.93) | 4.78 (-3.30 – 12.88) | -3.22 (-18.59- 8.60) |
| **SE** | 2.75 | 1.94 | 28.13 | 3.44 | 354.88 | 4.12 | 8.14 |
| **P-value** | 0.786 | 0.995 | 0.167 | 0.535 | 0.331 | 0.262 | 0.701 |
| ***SLC5A2***  **rs9934336** | -5.53 (-12.20 - 1.12) | -2.99 (-7.83– 1.83) | 16.86 (-57.93- 91.66) | -4.90 (-14.94 – 1.45) | 1514.84 (295.92 -2733.75) | -5.53 (-17.98 – 6.92) | -8.68 (-29.59 – 22.58) |
| **SE** | 3.40 | 2.46 | 38.16 | 4.08 | 621.91 | 6.35 | 12.93 |
| **P-value** | 0.123 | 0.242 | 0.666 | 0.245 | 0.045* | 0.396 | 0.518 |
| ***SLC5A2***  **rs9927250** | 1.71 (-4.91- 8.34) | 0.38 (-4.29 – 5.07) | -53.08 (-124.90- 18.73) | 1.59 (-10.99 – 9.52) | -84.23 (-1127.42 – 958.97) | -10.11 (-19.68 – 0.55) | 5.02 (-14.16 – 21.79) |
| **SE** | 3.38 | 2.39 | 36.64 | 4.90 | 532.25 | 4.87 | 9.06 |
| **P-value** | 0.619 | 0.873 | 0.173 | 0.749 | 0.878 | 0.053 | 0.592 |
| ***UGT2B4***  **rs1080755** | -6.11 (-11.96- 0.26) | -3.14 (-7.49 -1.20) | -24.65 (-95.09 -45.78) | -5.38 (-14.84-3.25) | -540.46 (-1409.21 – 328.28) | 12.06 (2.44 - 5.49) | 0.67 (-27.18 -20.56) |
| **SE** | 2.98 | 2.21 | 35.94 | 4.37 | 443.25 | 4.90 | 8.23 |
| **P-value** | 0.057 | 0.176 | 0.505 | 0.234 | 0.262 | 0.025* | 0.936 |
| ***SLC2A1***  **rs1385129** | 3.42 (-2.60- 9.45) | 2.86 (-1.30-7.03) | 41.53 (-23.92 -106.99) | 0.71 (-10.45-8.22) | 786.56 (-432.56 – 2005.67) | 0.16 (-9.15 – 9.48) | -5.22 (-19.61-12.84) |
| **SE** | 3.07 | 2.12 | 33.40 | 4.02 | 622.01 | 4.75 | 8.44 |
| **P-value** | 0.282 | 0.197 | 0.237 | 0.862 | 0.246 | 0.973 | 0.550 |
| **N** | 23 | 23 | 19 | 23 | 14 | 22 | 18 |

eGFR; estimated Glomerular Filtration Rate; FS, Fractional shortening; KCCQ, Kansas City Cardiomyopathy Questionnaire; LVEF, left ventricular ejection fraction; LVM, left ventricular mass; NT-proBNP, N-terminal pro–B-type natriuretic peptide; N, Number of patients involved in each analysis; PASP, Pulmonary artery systolic pressure; SE, Standard error

a; Model adjusted for Age, Sex, LVEF range, ACE/ARB/ARNI, BB, MRA; b, Model adjusted for Age, Sex, Atrial fibrillation, LVEF range; c, Model adjusted for Age, Sex, NYHA, Smoking status; d, Model adjusted for Age, Sex, LVEF range, Furosemide, Torsemide, MRA, ACE/ARB/ARNI; *, Significant p-value less than 0.05
